# A Deep-Learning Based Posture Detection System for Preventing Telework-Related Musculoskeletal Disorders

**DOI:** 10.3390/s21155236

**Published:** 2021-08-02

**Authors:** Enrique Piñero-Fuentes, Salvador Canas-Moreno, Antonio Rios-Navarro, Manuel Domínguez-Morales, José Luis Sevillano, Alejandro Linares-Barranco

**Affiliations:** 1 Architecture and Computer Technology Department, Escuela Técnica Superior de Ingeniería Informática—Escuela Politécnica Superior, University of Seville, 41012 Seville, Spain; epinerof@us.es (E.P.-F.); scanas@us.es (S.C.-M.); arios@us.es (A.R.-N.); mjdominguez@us.es (M.D.-M.); alinares@us.es (A.L.-B.); 2 I3US: Research Institute of Computer Engineering, University of Seville, 41012 Seville, Spain

**Keywords:** convolutional neural network, skeleton, posture, telework, e-health

## Abstract

The change from face-to-face work to teleworking caused by the pandemic has induced multiple workers to spend more time than usual in front of a computer; in addition, the sudden installation of workstations in homes means that not all of them meet the necessary characteristics for the worker to be able to position himself/herself comfortably with the correct posture in front of their computer. Furthermore, from the point of view of the medical personnel in charge of occupational risk prevention, an automated tool able to quantify the degree of incorrectness of a postural habit in a worker is needed. For this purpose, in this work, a system based on the postural detection of the worker is designed, implemented and tested, using a specialized hardware system that processes video in real time through convolutional neural networks. This system is capable of detecting the posture of the neck, shoulders and arms, providing recommendations to the worker in order to prevent possible health problems, due to poor posture. The results of the proposed system show that this video processing can be carried out in real time (up to 25 processed frames/sec) with a low power consumption (less than 10 watts) using specialized hardware, obtaining an accuracy of over 80% in terms of the pattern detected.

## 1. Introduction

Year 2020 became a turning point worldwide, forcing the entire population to make changes in their daily habits due to the pandemic, in which we are still immersed. Among these changes, those that concern employment have a special repercussion: many jobs were lost in the last year, which was a tragedy for the world economy. However, even among the population that kept their jobs, there have been substantial changes in the way they work, leading to a notable increase in teleworking [1].

According to the Eurofund study performed in July 2020 [2], 33.7% of EU employees work at home full-time, while an additional 14.2% of EU employees work at home part-time. In 2018, less than 5% of EU employees were full-time teleworkers (and less than 10% were part-time teleworkers) [3]; therefore, the impact of the pandemic led to an increase of more than 650% among full-time teleworkers. This study also reveals that almost half of these employees have never teleworked before.

On the other hand, working with a computer for many hours a day can cause injuries mainly to the spine, but also to the shoulders, neck, arms and/or wrists; those injuries are usually caused by incorrect postures in front of the computer. These problems are documented in multiple studies [4,5,6,7] and reveal the need to maintain good postural hygiene to avoid these injuries. Moreover, the probability of adopting wrong postures increases when most of the latest teleworkers have never worked this way before.

We can define the posture as the position that each person adopts when performing his/her activities, when walking, sitting, standing or sleeping. Regarding the term postural hygiene, it can be defined as the set of rules that aims to maintain the correct position of the body, either in stillness or in movement, and thus avoiding possible injuries. A proper posture maintains symmetrical body alignment around the head–spine–pelvis axis with the total weight distributed equitably, while a bad posture does not respect this alignment. Among the factors that affect posture, we can enumerate some of them: genetic and anatomical factors, mood, tension, stress, sport, overweight, sedentary lifestyle, bad nutrition, physiological state of the musculoskeletal system, excessive muscle activity and, last but not least, the objects that are used or carried on a day-to-day basis (bed, sofa, bags, work chair, etc.).

So, according to the previous explanations, the continuous use of the work chair in front of a computer can provoke asymmetries in the body alignment that, finally, may lead to spinal injuries such as hyperlordosis (very marked curvature of the spine in the lower back), hyperkyphosis (increase in the anterior curvature of the spine vertebral and dorsal), scoliosis (structural malformation of the spine) or rectifications (reduction of normal curvature of the spine) [8]. That is why recommendations on postural hygiene when working in a chair are important to take into account [9].

To verify the correct spine posture while the user is sitting on a chair, the parameters that are usually used measure the angle at which the spine, shoulders and neck are positioned and moved. In this way, it is useful to measure certain parameters, such as the lateral back bend, back flexion/extension, shoulder adduction/abduction, lateral neck bend, and neck flexion/extension, among others [10]. The recommended angles for those parameters used to measure the correct postures have been detailed in ergonomic and biomechanic studies [11,12], obtaining four ranges of motion zones (0 to 3), according to the strain put on the joints. This range study is summarized in Table 1.

As can be observed in Table 1, each parameter is divided into four ranges, according to the danger to the joints when maintaining those angles over a long period of time. Movements inside Zone 0 have a very low chance to damage the joints; these chances increase for Zone 1 (low), Zone 2 (medium) and Zone 3 (high). These ergonomic studies offer the possibility of developing recommendation systems in order to help the user to correct his/her posture; in fact, there are several works that obtained user’s posture information by using a wide variety of sensors and systems [13,14,15,16,17,18]. However, only a few of them indicated recommendations to the user and, moreover, the information obtained was processed (or studied by the specialist) offline and do not provide any information to the user in real time [19,20,21,22].

On the other hand, to correct incorrect posture habits, the current procedures include an assessment by the specialist and an action plan based on physical exercises and adaptation of the workspace [23]. Although this process is effective, on many occasions, the specialist is consulted long after, when there is already some damage to the neck, shoulders or spine.

Because of that, the early detection of bad posture before they cause damage to the user is crucial. In this area, the occupational risk prevention services of most companies usually conduct courses for employees to adapt their posture habits in front of the computer. However, with the 2020 epidemiological crisis, there was a massive migration of workers from the company buildings to their homes to telework, so the control of the employees’ workspace and posture habits was completely lost. This problem makes it more important to devise mechanisms capable of automatically detecting employees’ posture and offering recommendations to avoid the health problems indicated above.

Regarding the tools and mechanisms used so far to develop recommendation systems and to prevent anomalies and/or physical problems, the use of Machine Learning (ML) and Deep Learning (DL) techniques has become very important. In recent years, these techniques can be found in multiple works related to the health field, both for medical image analysis (such as [24,25,26]) and to detect patterns through physiological sensors (such as [27,28,29]). Moreover, some of them are related to detecting the correct posture in certain circumstances to avoid joint pain and severe bone diseases (such as [30,31,32]).

In addition, there are some works, developed in recent years, that are focused on the detection of the user’s skeleton through video processing [33,34]. These works usually use DL-based processing techniques with convolutional neural networks (CNN) to estimate the situation of the user’s various joints. With these previous works, it is feasible to design a skeleton detection and tracking system using CNN and, with this information, to design a recommendation system, aiming for the proper posture of the worker in front of the computer. However, video processing using CNNs requires significant computational resources to provide a real-time response. It would be much more comfortable and transparent for the worker if the system was integrated in the monitor (both the vision sensor and the processing system).

Thus, in this work, we aim to design, develop and test a skeletal detection system using CNNs that subsequently estimates the user’s posture and makes postural recommendations for the neck, shoulders and arms. This system is tested in real time, using various embedded systems, to measure both its effectiveness in detection and its response in real time as well as its energy consumption.

The rest of the manuscript is divided as follows: first, in the Materials and Methods Section, the characteristics of the posture detection system, the skeleton processing procedure and the system evaluation mechanism are presented. Next, the results after testing the system and evaluating the different hardware platforms are detailed and explained in the Results and Discussion Section. Finally, conclusions are presented.

## 2. Materials and Methods

**Figure 1:**
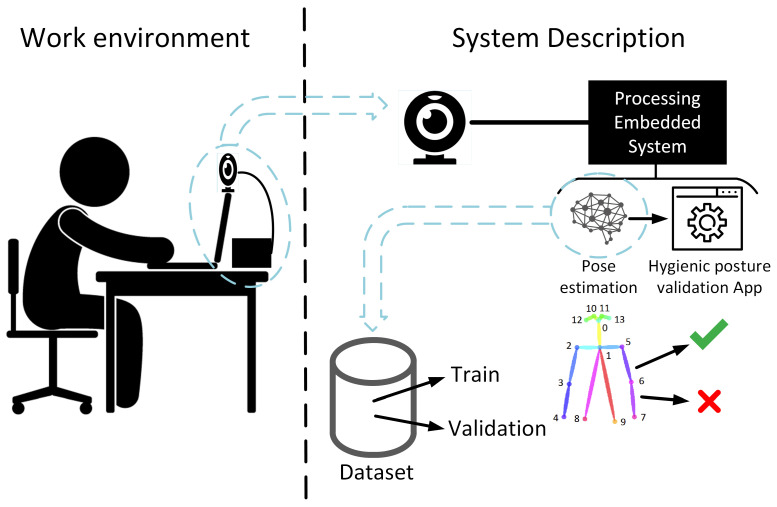
Application environment and global system overview.

The system is prepared to be placed on the computer screen or on a holder on top of it. The image acquisition device, a webcam or other type of camera, has to be pointing at the person to be able to capture them from the head to arms. This device is connected to an embedded system where the video is processed in two steps: first, the user’s joints’ positions are estimated, using a neural network classifier, and then the user’s whole posture is processed and validated in order to provide recommendations attending to some of the parameters described in Table 1. In Figure 1, a global system overview is presented.

The most computationally expensive task in the proposed system is the execution of the neural network that estimates the user’s joints. This type of algorithm needs sufficiently powerful hardware to run smoothly. Therefore, in this work, several embedded systems are evaluated in terms of execution time, power consumption and efficiency. The neural network used in this work requires supervised training, which means that it needs a training phase in which its internal parameters are adjusted to learn a specific task. This process is performed outside the embedded system due to its high computational workload, but the real-time classification is evaluated in the embedded system.

Summarizing, the full system has two main blocks: the first one is the skeleton estimator used to obtain the joints’ points; the second one is the recommendation system used to calculate the joints’ angles, classify them inside the four-range space and notify the position to the user. The first subsystem (skeleton estimator) is based on a pre-trained model that is embedded in a specialized hardware. So the information used to train this subsystem is based on a third-part dataset (COCO dataset) that contains 66,808 images with, at least, one person each. This dataset contains people of both genders, and all kinds of races and ages. The second subsystem (recommendation system) uses the information obtained from the output of the skeleton estimation subsystem (points’ list about the location of the joints) to calculate the different angles of the features used in this work and gives a notification to the user placed in front of the computer. The collected data for this work are used to test this second subsystem.

Next, the different parts of the processing system are described in depth. First, the embedded system that performs the posture detection process is detailed; secondly, the skeleton processing mechanism is presented; and, finally, the evaluation metrics used to test the system are shown.

### 2.1. Posture Detection System

To develop the posture detection system, a real-time pose estimation software called *TRT_Pose* was used [35]. This software is designed to run on NVIDIA devices, not only in desktop GPUs but also in embedded devices, that is, the NVIDIA Jetson family. So, this software is configured, executed and tested on different NVIDIA hardware devices that belong to that family. The main purpose of this evaluation is to obtain the performance metrics of those hardware boards in order to determine the best platform for this work. It is important to mention that, to obtain those evaluation metrics, the pose detection network was trained offline with the MSCOCO dataset [36], specifically on the *“Keypoints”* task (https://cocodataset.org/keypoints-2020 (last accessed on 20 June 21)). This public dataset contains 66,808 images with, at least, one person each. It contains people of both genders, and all kinds of races and ages; it is important to mention that the people tagged in this dataset are dressed in all kinds of attire (not just tight one), so bulkier garments are contemplated too, and the clothing factor does not affect the trained systems.

In Table 2, the hardware platforms used in this research are shown, detailing their computational performance, power consumption and other hardware characteristics and capacities. In this table, we can see the architectural differences of each device: from the most powerful one (NVIDIA GeForce GTX 1660 Super) to the less powerful (Jetson Nano). It is important to mention that the graphical board “NVIDIA GeForce GTX 1660 Super” is the only one that needs a desktop computer to be used (that is why columns “CPU” and “AI Performance” are not filled in Table 2); the rest of the hardware platforms are completely autonomous and do not need any other additional device to run. The main objective of using a classical GPU is to serve as a reference for the other devices.

As can be observed, the hardware devices are ordered from smallest (top) to largest (bottom) computing capacity, according to the GPU column. It is important to remark that, except for the first hardware device (Jetson Nano), the rest of them have more internal memory than the classic graphics card; this circumstance is due to the fact that, in a desktop computer, if the graphics card requires more memory, it usually uses the computer’s memory. However, for this work, this characteristic does not affect the boards’ performance.

Once the hardware devices used for the performance test are presented, the protocol used for capturing the user’s pose and obtaining the video streams used to test the system is detailed. As there is no available dataset that contains the information needed to this work (to the authors’ knowledge) we collected around 80 videoclips from 5 different users performing working tasks in front of a computer.

To capture the frames from each video stream, a webcam was placed on the computer’s screen frame, calibrated for each user at the same height as the line of sight. Each user was sitting on a height-configurable chair in front of the computer (so a full vision of the upper side of the human body is obtained) and he/she was instructed to compose a few lines in a text document. As we needed incorrect user’s poses to test our system, each user was ordered to perform the actions in different ways: first, those actions were performed in his/her natural pose; second, the user was forced to adopt an incorrect neck pose; third, the user was forced to adopt an incorrect shoulder balance; and, fourth, the user was forced to adopt an incorrect arm elevation pose.

**Figure 2:**
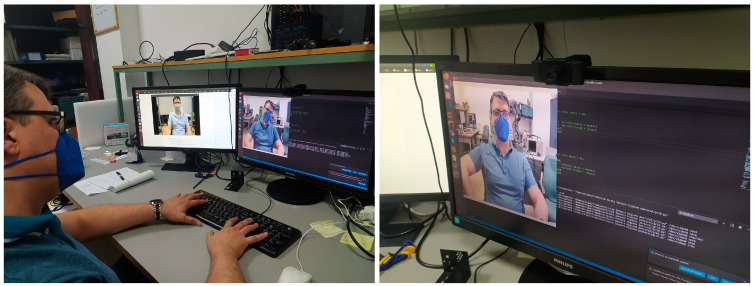
Setup with the webcam on the screen monitor.

The collected dataset was analyzed one by one by selecting several frames from each video clip to obtain the ground truth about the user pose adopted in each video. So, the entire dataset was divided into three subsets: neck poses, shoulder poses and arm poses. Additionally, each subset was labeled from range 0 to range 3, according to the degrees of variation adopted by the user on each video clip (the thresholds are shown in Table 1), which was analyzed offline. Some captures about the system setup when capturing the dataset’s video clips can be observed in Figure 2.

The final classification results of the system corresponds to the percentage of coincidence of the ranges estimated by the system in real time with respect to the established labels. Next, the skeleton processing stage, which allows to obtain the user’s joint points in real time, is described.

### 2.2. Skeleton Processing

As mentioned in the previous section, for the real-time skeleton detection in this work, we used the software called *TRT_Pose*. Specifically, we used a modified version of the original repository, which is called *TRT_Pose_Demo* [37].

The processing framework is based on a CNN model implemented in Pytorch [38], whose name is *ResNet* [39]. More concretely, we used a modified version of that model called *ResNet18* [40,41]. The benefits of using a “ResNet” arise with an idea similar to long short-term memory (LSTM) networks (recurrent neural networks) in which the temporal factor is taken into account to perform the classification. “ResNet” makes use of internal connections between layers that are not contiguous to communicate information from later (or earlier) moments in time with a specific moment. In this way, to perform the classification, not only can the information of the current frame be taken into account, but also the information of subsequent and/or previous frames (using connections similar to the bypasses that can be seen in the internal pipeline of a processor). About the variant *ResNet18*, the end number indicates the number of layers of the model.

**Figure 3:**
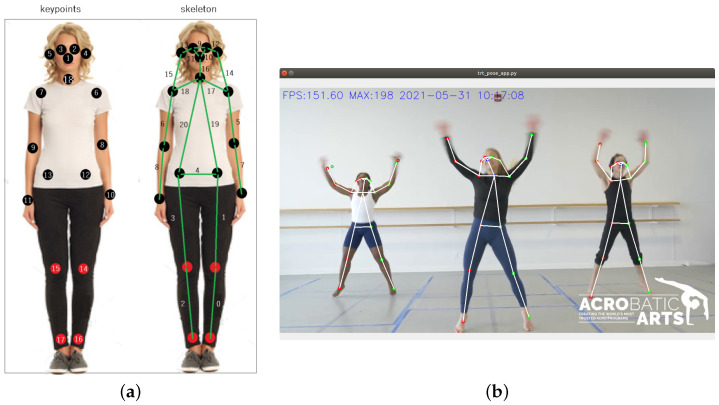
Output of the first subsystem: (**a**) structure and location of the skeleton’s keypoints that represent the joints; (**b**) execution example.

The final result of the deep learning model is a set of points related to the user joints detected, being able to detect more than one user in each frame. For this model, an user skeleton is defined by 18 points (this number is not related to the *ResNet* variant, as it is just a coincidence); the location of these points determines the person’s adopted pose. A global vision of the 18 points that this model detects, and their locations placed over the original image, is shown in Figure 3a.

Figure 3b shows an example of the real-time execution of the skeleton estimation system. As can be seen in both figures, the shape or pose of a person is delimited by the 18 points indicated above connected to each other in a specific way.

Once the skeleton estimation model task is defined, it should be noted that, although the network is trained and defined in PyTorch, a series of optimizations are performed prior to the execution of the model in real-time hardware (pre-processing step) for the new NVIDIA platforms that have tensor cores. So, the network previously passed through an optimization process that converts it for its execution on those specialized hardware. With these optimizations, besides allowing to be executed in that hardware, the data representation model is changed from a 32-bit Floating Point to a 16-bit Floating Point number representation.

Those optimizations are included because it is intended that the execution in the different embedded systems is as efficient as possible, always looking for the lowest possible consumption.

The performed pre-processing consists of converting the model trained in PyTorch format to a format compatible with NVIDIA’s *Tensor RT* [42] (TRT) library, which is designed with the aim of accelerating the neural network estimation process. For this purpose, a tool called *Torch2TRT* [43] was used, which precisely converts the trained PyTorch model into a version compatible with the Tensor RT libraries. The number precision reduction from 32-bit floating point to half precision (Float16) during optimization reduces the computation time as well as energy consumption, at the cost of reducing the final accuracy of the model by less than 2%, according to previous studies [44]. This is a technique widely used when tuning models for execution in embedded and/or edge-computing systems since, usually, the loss of accuracy is minimal compared to the gain in performance.

Finally, in the last subsection, the processing and metrics performed to evaluate the implemented system are detailed.

### 2.3. System Evaluation

In this section, we detail how our system recognizes a correct posture, how we are going to evaluate the ground-truth postures collected and the metrics used to evaluate the performance of the system.

#### 2.3.1. Posture Recognition

**Figure 4:**
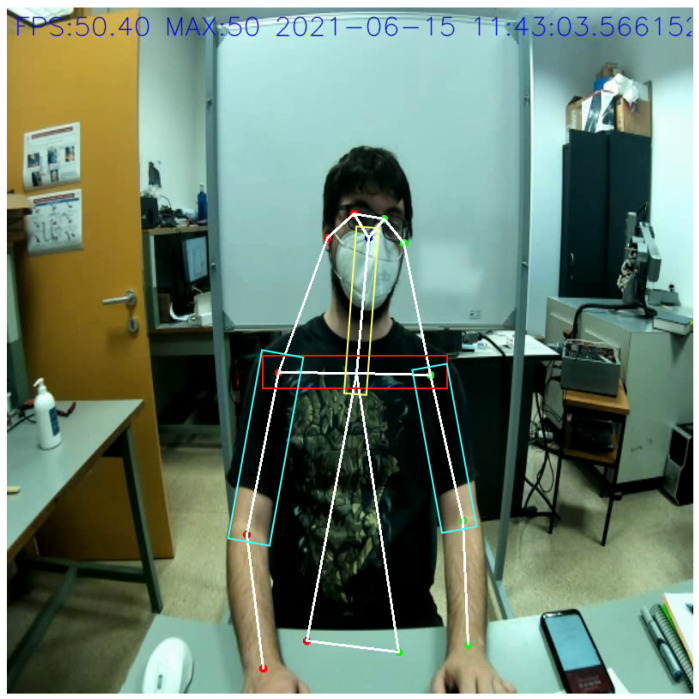
Evaluable parameters from test dataset. Parameters are marked over the CNN output skeleton with different colors: neck lateral bend (yellow), shoulder alignment (red) and arms abduction (cyan).

Given a posture, there are multiple ways in which we can assess whether or not it is correct. This ultimately means that there are a lot of different factors that apply, as we can see in [10] and in Table 1, so evaluating a specific posture is a non-trivial task and can be solved using different methods and metrics. To keep things simple while at the same time being able to solve the problems related to the current teleworking needs, we have decided to analyze three aspects of the posture: the neck lateral bend, the shoulders alignment and the arms abduction (also related to the shoulders). These parameters can be seen marked in Figure 4. Each of these parameters has four possible zones in which they can be, ranging from good posture (healthy) to bad posture (very unhealthy) progressively. The first zone, denoted as “Range 0” in Table 1, corresponds to the best posture and therefore the healthiest, while the fourth zone, “Range 3” in the table, corresponds to the worst possible posture. With this in mind, we take into account the already mentioned parameters for each image evaluated and classify them using Table 1’s criteria.

#### 2.3.2. Posture Evaluation

Once we know how to recognize a good posture, we need to apply it to the postures collected during the work. For that, we can take advantage of the skeleton processing system’s output to evaluate the angle of rotation of each evaluable parameter and classify it as described before. In order to do this, that output needs to consist not only of a video with the estimated skeleton, but also a collection of data that gives details about which keypoints are estimated at each step of time. Given this information, we can relate the timestamp of a specific frame with the timestamp of the data collection described earlier to obtain the pertinent keypoints and perform the required calculations. This is an offline procedure performed to manually tag the dataset collected. In this work, the accuracy of the collected dataset is evaluated, performing a proof of concept of the final system. After evaluating it, in future works, it will be adapted to a real-time application.

Calculations are performed, taking into account the following: we only have two points per parameter and we need to calculate the angle of the line expressed by those two points to classify it inside the mobility range (ranges detailed in Table 1). The points used for evaluating each parameter are detailed in Table 3.

These two points form the part of the skeleton that was estimated by the CNN and are taken from the data collection mentioned before, while the angle calculation is performed with the *atan2* function. This function is evaluated as shown in Equation (1).

(1) $$atan2\left(y,x\right)=\left\{\begin{array}{cc} arctan(\frac{y}{x})\; & \mathrm{if}\;x>0\\ arctan(\frac{y}{x})+\pi \; & \mathrm{if}\;x<0\;\mathrm{and}\;y\geq 0\\ arctan(\frac{y}{x})-\pi \; & \mathrm{if}\;x<0\;\mathrm{and}\;y<0\\ +\frac{\pi }{2}\; & \mathrm{if}\;x=0\;\mathrm{and}\;y>0\\ -\frac{\pi }{2}\; & \mathrm{if}\;x=0\;\mathrm{and}\;y<0\\ \mathrm{undefined}\; & \mathrm{if}\;x=0\;\mathrm{and}\;y=0 \end{array}\right.$$

#### 2.3.3. Metrics

The main goal of the system is to classify a series of angles extracted from the keypoints shown in Table 3 estimated by the CNN. So, in order to evaluate the results obtained after the estimations, we compare the tag stored during the recording process with the results obtained after obtaining the mobility ranges extracted with the degrees resulting from Equation (1). Therefore, we may define the accuracy of the system, *A*, as the ratio between the correct predicted outputs and the total predicted outputs per zone or class, denoted by Equation (2). TP and TN mean *true positives* and *true negatives*, while FP and FN mean *false positives* and *false negatives*. These parameters compose the output of the system, and tell us how the system predicted each and every of its inputs, whether they were correct or not. For each class, the information concerning the classification results is extracted by looking at that class independently. Another way is to see the global ranking only, but that provides much less information.

(2) $$A_{total}=\frac{TP+TN}{TOTAL},\;where:\;TOTAL=TP+TN+FP+FN$$

After presenting the implemented system and how it will be evaluated, in the next section, the results obtained by the pose detection system are detailed.

## 3. Results and Discussion

In this section, we detail the dataset collected and the performed results of the postural study, as well as a comparison with other related studies.

### 3.1. Collected Data

For this work, and after a second data collection process, 12 people have participated to record the dataset used. A series of recordings were performed for each person, in which each one held a different posture for each recording, so that we may have a variety of data to process and classify. Finally, a total amount of 151 correct recordings were made, and a total of 148 frames were processed (some of them were discarded due to recording errors).

The final dataset contains information about 12 users in their early 20’s to early 50’s, from both genders and both dominant hands.

The aim of this work is to evaluate the system, which is why random frames were extracted from the video recordings instead of evaluating the whole videos (this way, the tag process and the evaluation are more accurate); however, the system can work in real time, processing frame by frame.

### 3.2. Results

The system achieved a total accuracy of 84.1%, as can be seen in Table 4. As can be observed, the accuracy of each parameter is similar, obtaining the worst result (81.4%) with “Left Arm Abduction” and the best result (87.7%) with “Shoulder Alignment”. It is important to remark that the dataset includes subjects of both genders, ages from early 20’s to early 50’s, both dominant hands and different clothes.

**Figure 5:**
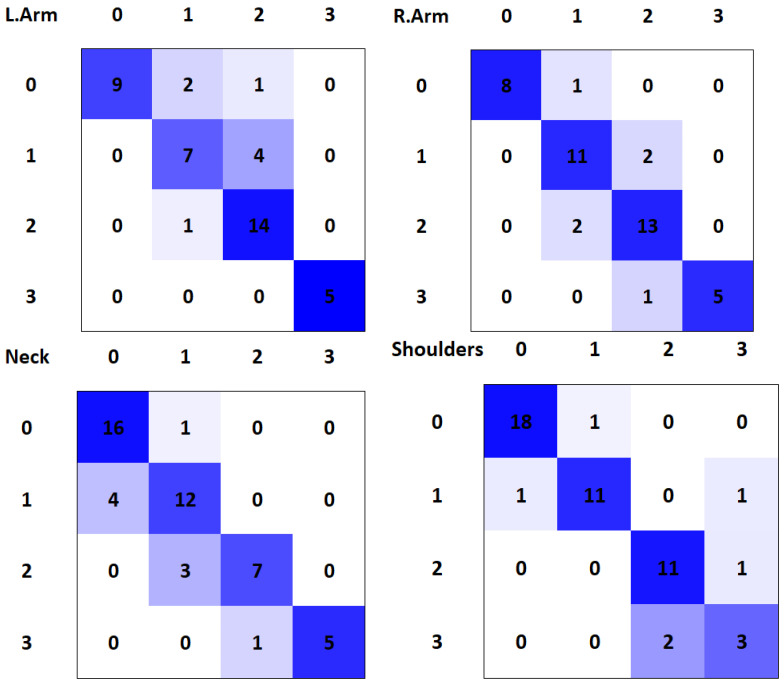
Confusion matrices for right arm abduction (**top left**) left arm abduction (**top right**) neck lateral bend (**bottom left**) and shoulder alignment (**bottom right**).

The system’s predictions per class can be visualized in Figure 5, which are the confusion matrices for each class. In them, the proportion of predictions against ground truth values is displayed, being that the main diagonal of the matrix is the correct predictions of the system. With these matrices, TP,TN,FP and FN values can be obtained per class, while also having a compact representation.

It can be observed that the best results, as shown in Table 4, are presented for the “Shoulder Alignment” parameter (Figure 5-top-right). On the other hand, we can observe two peculiar cases: one of them in “Left Arm Abduction”, where a range 0 case is classified as range 2; and the other one in “Shoulder Alignment” parameter, where a range 1 case is classified as range 3. Apart from those two errors, the rest of the errors occur from one range to a contiguous one.

Moreover, knowing that the dangerous ranges are 2 and 3, a critical error would be produced if a dangerous pose (range 2 or 3) is classified as a safe pose (range 0 or 1). Evaluating only those two classes (critical and safe) for each parameter, the accuracy is improved. The results obtained after this group can be observed in Table 5.

As can be observed in Table 5, the mean accuracy in this case is 92.4%. The worst case is shown with “Left Arm Abduction”, obtaining a 86% accuracy; the best case is obtained for “Shoulder Alignment” with a 97.9% accuracy.

The dataset collected is not very large, but there is a great deal of variability among the participants. For this reason, an exhaustive study of the testing dataset was carried out, using bootstrap re-sampling. In this case, we have generated 15 random testing subsets, each one of them taking 50% of the samples from the full dataset. Each of these 15 subsets was evaluated independently, obtaining the classification accuracy for each of the parameters studied (neck, shoulders, right arm and left arm) and testing the 4-classes classification (range 0–3) and the 2-classes classification (ranges 0–1 and 2–3). The results obtained can be seen in Table 6.

As can be seen in Table 6, the evaluation by re-sampling the dataset confirms some of the points previously indicated in the overall evaluation. On the one hand, the parameter for which the worst accuracy is obtained is the left arm, although, in this evaluation, a greater closeness with the right arm is observed (difference of less than 5%), which means that the detection of the position of the arms seems to be more complex than the detection of the remaining parameters. Secondly, it is confirmed that the most accurate parameter is the shoulders, obtaining a significant difference with respect to the others. Finally, neck detection is the second parameter in terms of classification perception. In short, qualitatively, it is similar to the global results but with slightly varying classification percentages (which depends on the random sample that was classified in each case).

With respect to the standard deviation after the classification of the 15 subsets, it can be seen that the shoulders are the ones with the lowest deviation, while the remaining parameters have a similar standard deviation.

If we consider the second part of Table 6, where results regarding the classification for the 2-class case are presented instead of the 4-class case, significant improvements can be observed. However, the relative accuracy remains the same, with the shoulders being the best ranked parameter, the neck the second-best ranked parameter and, in last position, the arms (with a slight variation between the right and the left arm, which is the one with the worst accuracy). As for the standard deviation, they are significantly reduced, except in the case of the left arm, which maintains practically the same value.

Therefore, with these data, we can assume that the complete dataset has consistent results and that all the samples follow the same pattern by having a standard deviation in all cases lower than 5%. Likewise, the fact that the classification with two classes yields much better results than those obtained with four classes is corroborated.

### 3.3. Performance Tests

**Figure 6:**
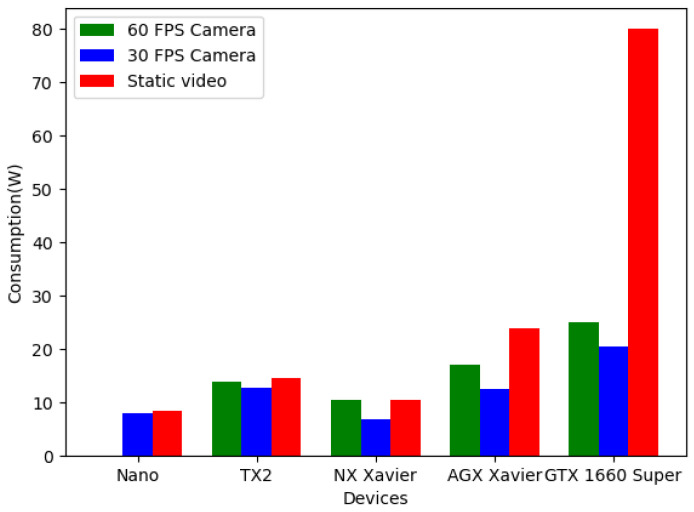
Consumption in watts for each device and test.

**Figure 7:**
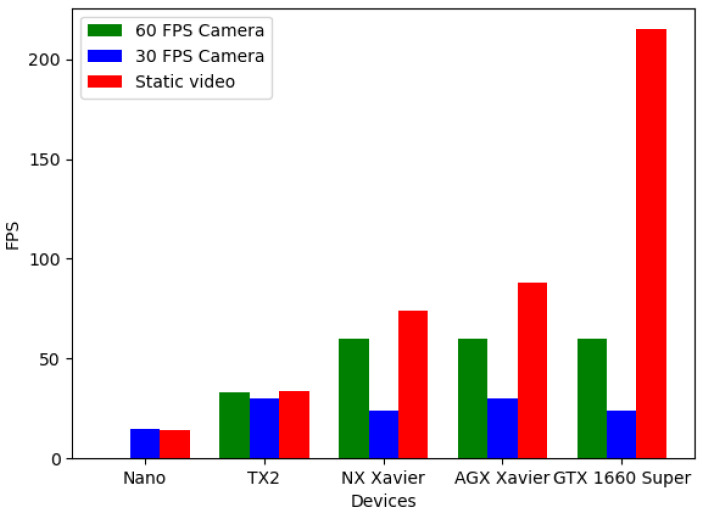
Achieved frame rates per device and test.

**Figure 8:**
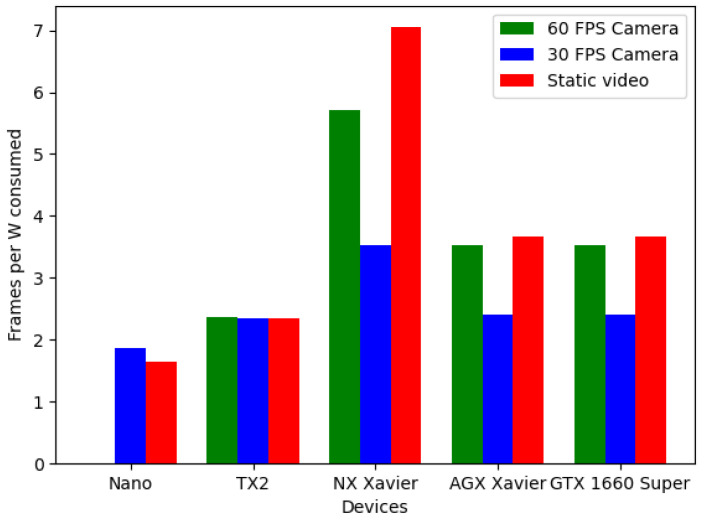
Ratio frame processed per watt consumed for each device and test.

A performance study on the different hardware platforms presented was also conducted in parallel. In it, we measured the consumption and total throughput of every device while running the CNN, so that we may give at least a recommendation about which device would be a good fit for setting up a system, such as that presented, taking into account the already mentioned parameters. The results can be seen in Figure 6, Figure 7 and Figure 8.

To be able to measure such parameters, three tests were designed. In two of them, the CNN’s input is a real-time camera but one has a frame rate of 30 FPS and the other one has 60 FPS. The third test consists of the classifying of an offline video, which was made using only one single repeated frame; this way, the memory and other aspects would not be a bottleneck and so we could obtain a significant reference value of maximum throughput.

As we can see, almost all the devices presented would be a good fit; however, given the obtained results, a viable recommendation for building a system like the one presented would be the NX Xavier, as its efficiency is the maximum achieved, with 7 frames processed per watt consumed in the offline video test and 6 for the 60 FPS camera test.

## 4. Conclusions

Due to the pandemic situation of year 2020, the number of teleworkers has increased significantly. Such an amount of hours in front of a computer in a work space not properly adapted for that workload can lead to postural problems for the employee. That is why a system like the one proposed in this work is necessary.

After evaluating several hardware platforms and testing the system with multiple users, the results obtained in this study show that a postural recommendation system is more than viable, using the resources presented. Results showed a posture detection accuracy over 80% for the 4-class original problem, and more than 90% for the 2-class classification system. On the other hand, the hardware platforms tested allows the system to run in a real-time environment with low power consumption requirements. With these two points in favor, we can conclude that the system can work completely autonomously and without the intervention of a computer, providing information in real time.

Although this work was implemented and tested in a laboratory environment with a not very extensive dataset, the results are very promising. Moreover, this only reinforces the fact that there is still quite a lot to gain from automating and detailing the process. Future research may include real-time video processing with an automatic recommendation system, a collection of incorrect poses during real work sessions to improve overall postures, or even changes to the CNN used in order to obtain a more detailed pattern recognition that would allow for the checking of other parameters, such as the spine, which requires more than a single straight segment to be evaluated.

The metrics obtained in the results should be taken as further confirmation of the hypothesis, as the system is able to recognize correctly most of the inputs it is fed. A bigger study with more people and/or parameters is also interesting, as it would serve as even more proof that this kind of work is viable.

## Figures and Tables

**Table 1:** Range (in degrees) of motion zones (0 to 3) for some of the parameters used to quantify the body posture.

Parameter	Range 0	Range 1	Range 2	Range 3
Back—Lateral bend	0–5	6–10	11–20	21+
Back—Flexion	0–10	11–25	26–45	46+
Back—Extension	0–5	6–10	11–20	21+
Shoulder—Alignment	0–5	6–10	11–20	21+
Shoulder—Arms adduction	0–5	6–12	13–24	25+
Shoulder—Arms abduction	0–13	14–34	35–67	68+
Neck—Lateral bend	0–5	6–12	13–24	25+
Neck—Flexion	0–9	10–22	23–45	46+
Neck—Extension	0–6	7–15	16–30	31+

**Table 2:** NVIDIA Jetson devices comparison. Data obtained from https://developer.nvidia.com/ (last accessed on 28 May 2021).

Device	GPU	CPU	Memory	Consumption (Min/Max)	AI Performance (TFLOPS)
Jetson Nano	128-core Maxwell GPU 921 MHz	Quad-Core ARM Cortex-A57 1.43 GHz	4 GB 64-bit LPDDR4 Dual Channel 1.6 GHz (25.6 GB/s)	5 W/10 W	0.472
Jetson TX2	256-core Pascal GPU 1.36 GHz	Dual-Core Denver2 64-Bit CPU and Quad-Core ARM Cortex-A57 2 GHz	8 GB 128-bit LPDDR4 1.866 GHz (59.7 GB/s)	7.5 W/15 W	1.33
Jetson Xavier NX	384-core Volta GPU 48 Tensor Cores 1.1 GHz	6-core Carmel ARMv8.2 64-bit CPU 1.9 GHz	8 GB 128-bit LPDDR4 x 1.6 GHz (51.2 GB/s)	10 W/15 W	21
Jetson AGX Xavier	512-core Volta GPU 64 Tensor Cores 1.4 GHz	8-core Carmel Armv8.2 64-bit CPU 2.3 GHz	32 GB 256-bit LPDDR4 x 2.133 GHz (136.5 GB/s)	10 W/30 W	32
NVIDIA GeForce GTX 1660 Super	1408-Core Turin GPU 1.785 GHz	N/A	6 GB GDDR6 192-bit 14 Gbps (336 GB/s)	10 W/125 W	N/A

**Table 3:** Points used to evaluate each parameter.

Parameter	Point 1 (x)	Point 2 (y)
Shoulder—Alignment	Left Shoulder	Right Shoulder
Right Arm adduction	Right Shoulder	Right Elbow
Left Arm abduction	Left Shoulder	Left Elbow
Neck—Lateral bend	Nose	Neck

**Table 4:** Accuracy results for each parameter.

	Right Arm	Left Arm	Neck	Shoulder	Mean
Accuracy	0.860	0.814	0.816	0.877	0.841

**Table 5:** Accuracy results for each parameter using a 2-class classifier: safe (range 0–1) and dangerous (range 2–3).

	Right Arm	Left Arm	Neck	Shoulder	Mean
Accuracy	0.906	0.860	0.938	0.979	0.924

**Table 6:** Bootstrap re-sampling evaluation with 15 random subsets.

	4 Classes				2 Classes			
**Set**	**Neck**	**Shoulder**	**R.Arm**	**L.Arm**	**Neck**	**Shoulder**	**R.Arm**	**L.Arm**
1	0.909	0.909	0.917	0.70	1	1	0.917	0.70
2	0.826	0.909	0.857	0.809	0.956	1	0.905	0.857
3	0.818	1	0.809	0.773	0.864	1	0.905	0.864
4	0.955	0.955	0.952	0.857	1	1	0.952	0.857
5	0.864	0.955	0.809	0.857	0.955	1	0.905	0.905
6	0.955	1	0.864	0.727	1	1	0.909	0.818
7	0.864	0.955	0.773	0.773	0.955	1	0.864	0.818
8	0.955	0.909	0.952	0.809	1	1	0.952	0.857
9	0.818	0.955	0.809	0.857	0.955	1	0.905	0.905
10	1	0.909	0.864	0.762	1	1	0.864	0.809
11	0.864	0.955	0.818	0.818	0.955	1	0.909	0.909
12	0.864	0.955	0.809	0.773	1	1	0.857	0.909
13	0.909	1	0.773	0.773	1	1	0.818	0.818
14	0.818	0.955	0.809	0.857	0.955	1	0.857	0.905
15	0.864	1	0.809	0.818	0.955	1	0.905	0.864
MIN	0.818	0.909	0.773	0.70	0.864	1	0.818	0.70
MAX	1	1	0.917	0.857	1	1	0.952	0.909
MEAN	0.885	0.955	0.842	0.797	0.969	1	0.895	0.853
STD	0.049	0.024	0.047	0.041	0.028	0	0.028	0.040
Improve					9.54%	4.76%	6.31%	6.94%
